# Concomitant splenic artery ligation has no preventive effect on left‐sided portal hypertension following pancreaticoduodenectomy with the resection of the portal and superior mesenteric vein confluence for pancreatic ductal adenocarcinoma

**DOI:** 10.1002/ags3.12545

**Published:** 2022-02-10

**Authors:** Kazuyuki Gyoten, Shugo Mizuno, Motonori Nagata, Takahiro Ito, Aoi Hayasaki, Yasuhiro Murata, Akihiro Tanemura, Naohisa Kuriyama, Masashi Kishiwada, Hiroyuki Sakurai

**Affiliations:** ^1^ Department of Hepatobiliary Pancreatic and Transplant Surgery Mie University School of Medicine Tsu Japan; ^2^ Department of Radiology Mie University School of Medicine Mie University School of Medicine Tsu Japan

**Keywords:** left‐sided portal hypertension, plenic artery ligation, splenic vein division, splenomegaly, thrombocytopenia, varices

## Abstract

**Background:**

Left‐sided portal hypertension (LSPH) caused by splenic vein (SV) division in pancreaticoduodenectomy (PD) with portal vein (PV) resection remains challenging. The current study aimed to investigate the efficacy of splenic artery (SA) ligation in preventing LSPH.

**Methods:**

One‐hundred thirty patients who underwent PD with PV resection for pancreatic ductal adenocarcinoma were classified into SV and SA preservation (SVP, n = 30), SV resection and SA preservation (SVR, n = 59), and SV resection and SA ligation (SAL, n = 41). The postoperative incidence of LSPH was assessed.

**Results:**

The incidence of variceal formation in SVP, SVR, and SAL were 4.8%, 53.2%, and 46.4% at 3 mo, 13.0%, 71.2%, and 62.5% at 6 mo, and 25.0%, 87.5%, and 87.1% at 12 mo, respectively. The rate was significantly higher in SVR at 3 and 6 mo (*P* = .001 and *P* < .001, respectively) and in SVR and SAL (*P* < .001) at 12 mo. Variceal hemorrhage occurred only in SVR (n = 4). The platelet count ratio at 3, 6, and 12 mo began to significantly decrease from 3 mo in SVR (0.77, 0.67, and 0.60, respectively; *P* < .001) and 6 mo in SAL (0.91, 0.73, and 0.69, respectively; *P* < .001). The spleen volume ratio also showed significant increase from 3 mo in SVR (1.24, 1.34, and 1.42, respectively; *P* < .001) and 6 mo in SAL (1.31, 1.32, and 1.34, respectively; *P* < .001). SVR and SAL were significant risk factors for variceal formation at 12 mo (odds ratio, 21.0 and 20.3, respectively).

**Conclusion:**

In PD with PV resection, SAL delayed LSPH but could not prevent its occurrence.

## Graphical abstract

The splenic vein division in pancreaticoduodenectomy with portal vein resection causes left‐sided portal hypertension (LSPH), which manifests as variceal formation and hemorrhage, thrombocytopenia and splenomegaly. Concomitant splenic artery ligation delayed but could not prevent development of LSPH; it did not cause variceal hemorrhage.

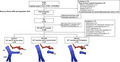

## 1 INTRODUCTION

Pancreaticoduodenectomy (PD) for pancreatic ductal adenocarcinoma (PDAC) commonly requires the resection and reconstruction of the portal vein (PV) / superior mesenteric vein (SMV) confluence. The splenic vein (SV) often needs to be resected to achieve tumor clearance or maximize the mobility of PV/SMV and is rarely reconstructed.^1^ The division of the SV can lead to left‐sided portal hypertension (LSPH), causing gastrointestinal variceal formation, thrombocytopenia, and splenomegaly.^1^, ^2^, ^3^, ^4^

To prevent postoperative LSPH, additional concomitant surgical procedures have been reported, with some surgeons claiming to have reconstructed the SV to PV/SMV, inferior mesenteric vein (IMV), or left renal vein.^5^, ^6^, ^7^, ^8^ However, this reconstruction is not simple because SV tends to be too long, with its long‐term patency being unknown. Conversely, splenic artery ligation (SAL) is a simple procedure for portal modulation.^9^, ^10^, ^11^, ^12^ Previously, we reported the efficacy of concomitant splenic artery resection (SAR) in preventing LSPH after PD with resection of the PV/SMV confluence.^2^ Therefore, we performed concomitant SAL to reduce the incidence of LSPH following PD with PV/SMV resection and SV division since September 2016. Here we aimed to determine the effect of PD‐SAL on LSPH incidence, paying attention to variceal formation, collateral development, platelet counts, and spleen volume.

## 2 METHODS

### 2.1 Patients

**FIGURE 1:**
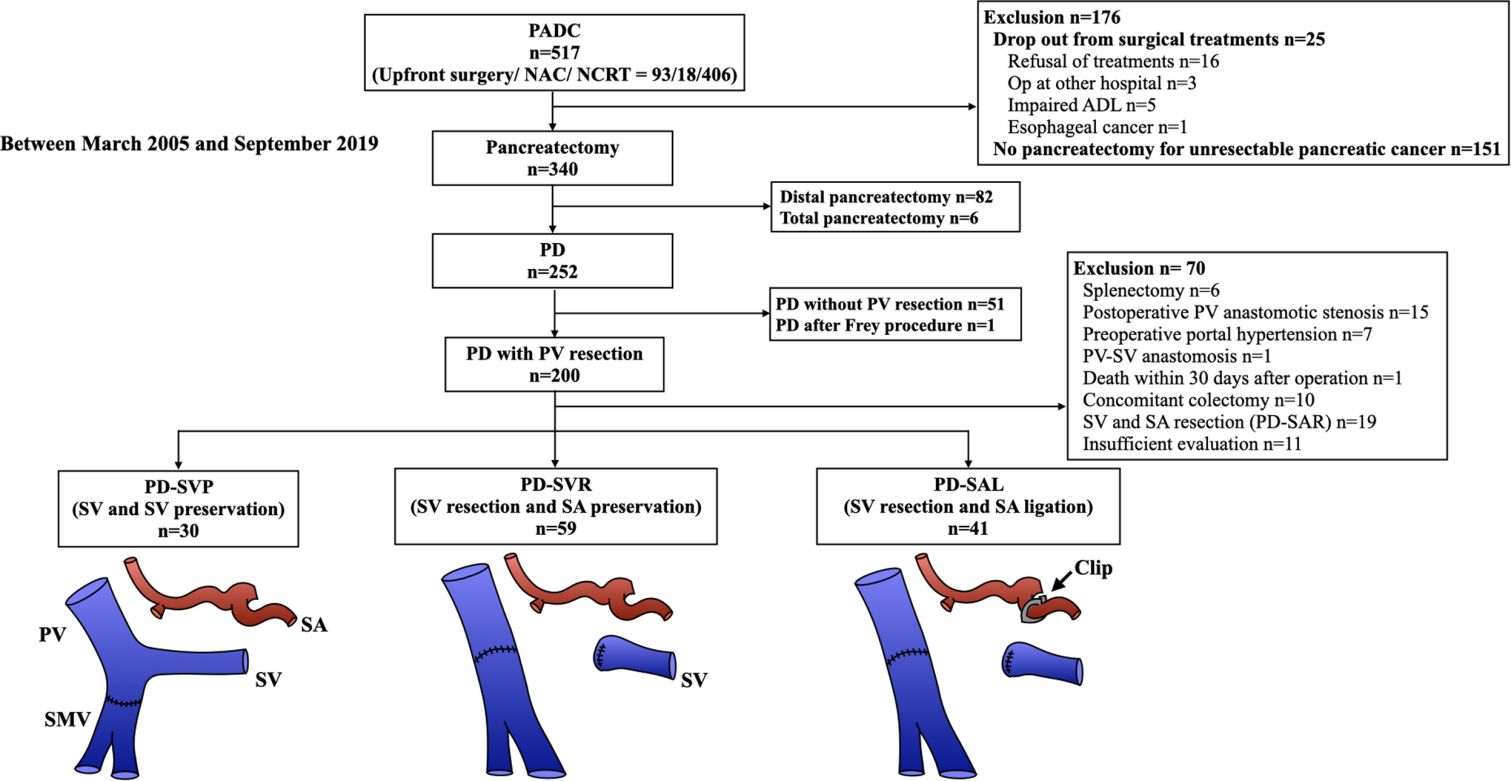
Treatment flow for pancreatic ductal adenocarcinoma. One‐hundred thirty patients who underwent PD with PV resection were enrolled in the present study. ADL, activities of daily living; DP, distal pancreatectomy; NAC, neoadjuvant chemotherapy; NCRT, neoadjuvant chemoradiotherapy; PD, pancreaticoduodenectomy; PDAC, pancreatic ductal adenocarcinoma; PV, portal vein; SA, splenic artery; SAL, SA ligation; SAR, SA resection; SV, splenic vein; SVP, SV preservation; SVR, SV resection; TP, total pancreatectomy

Between March 2005 and September 2019, 517 consecutive patients were diagnosed with PDAC using 64‐slice multidetector computed tomography (MDCT). Surgery was performed in 93 patients, 18 received neoadjuvant chemotherapy, and 406 received chemoradiotherapy (CRT).^13^ Among them, 200 patients underwent PD with PV/SMV resection. To evaluate the development of postoperative LSPH precisely, 70 patients were excluded: resection of both SV and SA (n = 19), preoperative portal hypertension due to PV and/or SV occlusion (n = 7), concomitant colectomy (n = 10), concomitant splenectomy (n = 6), intraoperative PV/SV anastomosis (n = 1), postoperative stenosis of PV/SMV anastomosis (n = 15), insufficient follow‐up (n = 11), and in‐hospital death within 30 d postoperative (n = 1). In this observational, single‐center retrospective study, the 130 patients who underwent PD with PV/SMV resection were enrolled and classified into three groups: SV and SA were both preserved (PD‐SVP) in 30, the SV was resected and SA preserved [PD‐SVR] in 59, and the SV was resected and SA ligated [PD‐SAL] in 41 (Figure 1).

### 2.2 Preoperative characteristics and surgical outcomes

We collected data on various preoperative factors such as age, sex, body mass index (BMI), maximum tumor size on CT, performance status, complete blood count parameters, albumin, carcinoembryonic antigen (CEA) and carbohydrate antigen (CA19‐9), spleen volume, the presence of preoperative chemotherapy or chemoradiotherapy, T and N factors according to the Union for International Cancer Control (UICC) 8th classification, and resectability of the tumor (classified into resectable, R; borderline resectable, BR; and unresectable, UR) according to The National Comprehensive Cancer Network guideline,^14^ based on the findings of MDCT as previously reported. We also evaluated surgical procedures and outcomes, including PD and subtotal stomach‐preserving PD (SSPPD), the left gastric vein (LGV) division or preservation including the confluence variant, the IMV division or preservation including the confluence variant, intraoperative blood loss, operative duration, degree of postoperative complications according to the Clavien–Dindo (C‐D) classification,^15^ pancreatic fistula according to the International Study Group on Pancreatic Fistula,^16^ the presence of pathological PV invasion (pPV), the achievement of curative resection (R0 resection), and postoperative hospital stay duration.

### 2.3 Surgical procedures

An anterior approach to the superior mesenteric artery (SMA) has been the standard resection technique in PD for PDAC.^17^ The middle colic vein (MCV) was resected in all cases for lymph node dissection. The anastomosis between PV and SMV was performed using a 6‐0 nonabsorbable running suture. An interposition graft was used only when a primary repair was not feasible for the anastomotic tension. When the SV, LGV, or IMV was involved in the tumor, they were divided and not reconstructed. Reconstruction of the digestive tract was performed using a modified Child method, end‐to‐side pancreaticojejunostomy, end‐to‐side hepaticojejunostomy, and end‐to‐side or side‐to‐side gastrojejunostomy. In SAL, the root of the SA was clumped using Hem‐o‐lock (Weck Surgical Instruments; Teleflex Medical, Morrisville, NC).

### 2.4 Assessment of LSPH

To assess the development of LSPH, newly developed digestive varices and collateral routes were evaluated at postoperative 3, 6, and 12 mo using enhanced MDCT by the radiologist (N.M.), who was not informed of patient characteristics or outcomes. Esophageal, gastric, pancreatic, and colonic varices were diagnosed when the dilated and beaded veins were detected within the submucosal layer of each organ compared with preoperative.^2^

Development of collateral pathways from the divided SV were diagnosed when the diameter of collateral routes became 1.5 times larger than the preoperative. Spleno‐renal and gastro‐renal shunts were evaluated as spleno‐systemic routes. Superior and inferior routes were evaluated as spleno‐portal routes according to a previous study.^18^ The superior route was defined as a pathway starting in the divided SV, following a superior and rightward direction through perigastric veins and LGV, and finally ending in the PV. The inferior route was defined as a pathway starting from the divided SV, joined to venous routes in the mesocolon through the omental arcade and/or the IMV, and proceeding in an inferior and rightward direction to end in the SMV.^3^, ^18^ Blood supply to the spleen after PD‐SAL was evaluated based on enhanced MDCT within postoperative 14 d.

Platelet count data were collected preoperative and at postoperative 3, 6, and 12 mo. The platelet count ratio was calculated as postoperative count divided by preoperative count. Postoperative thrombocytopenia of grade 2 or higher (less than 75,000/mL) based on the Common Terminology Criteria for Adverse Events Version 5.0 was also evaluated. The total spleen volume was estimated by tracing the spleen on each transverse CT image obtained at 2.0‐mm intervals. Spleen volume was measured preoperative and at postoperative 3, 6, and 12 mo. The spleen volume ratio was calculated as the postoperative volume divided by the preoperative volume. When patients did not undergo enhanced CT or a blood test at that time (with a margin of 1 mo at 3 and 6 mo, and 2 mo at 12 mo), they were excluded from each analysis. We stopped the evaluation of LSPH after patients developed PV/SMV occlusion, underwent portal modulation for LSPH, or succumbed to recurrent PDAC or other diseases.

The study protocol was approved by the Medical Ethics Committee of Mie University Hospital (No. H2019‐070), and informed consent was obtained from each participant on an opt‐out basis.

### 2.5 Statistical analyses

All continuous values are presented as median (range). Continuous variables were compared using the Mann–Whitney *U* test or Kruskal–Wallis test. Categorical variables were compared using Pearson's chi‐squared or Fisher's exact test, as appropriate. The changes in postoperative platelet count and spleen volume ratios were evaluated using the Freidman test. Stepwise forward multiple logistic regression analysis of risk factors contributing to variceal formation postoperative was performed using perioperative variables, which showed *P* < .25 in univariate analysis. Statistical analyses were performed using the IBM rel. 2016 (IBM SPSS Statistics for Windows, v. 24.0; IBM, Armonk, NY, USA). *P* < .05 was considered statistically significant.

## 3 RESULTS

### 3.1 Patient background and surgical outcomes

Table 1 shows comparisons of patient background and surgical outcomes among the groups. The preoperative CEA level, the ratio of CRT to upfront surgery, blood loss level, and postoperative hospital stay were significantly highest in the PD‐SVR group. LGV or IMV preservation was significantly higher in the PD‐SVP group than in the other groups. There was no significant difference in postoperative complications of C‐D grade IIIa or higher and pancreatic fistula.

**TABLE 1:** Comparison of patients’ background and surgical outcomes. [Correction added on 02 March 2022, after first online publication: some data in tables has been changed to bold and some data in column 1 has been indented]

Perioperative variables	PD‐SVP n = 30	PD‐SVR n = 59	PD‐SAL n = 41	*P*
Age	69 (51‐83)	65 (41‐83)	69 (48‐85)	0.173
Male/Female	14/16	41/18	21/20	0.062
BMI, kg/m^2^	20.5 (15.2‐27.1)	20.5 (15.2‐28.3)	22.6 (14.0‐27.0)	0.052
Performance status 0/1/2/3	21/7/2/0	36/22/1/0	30/9/1/1	0.286
Maximum tumor size on CT, mm	23.0 (13.1 ‐38.7)	25.7 (11.2‐45.9)	22.6 (10.6‐44.0)	0.202
**CEA, ng/mL**	2.9 (1.5‐9.6)	**4.1 (1.3‐22.8)**	**2.7 (0.9‐9.2)**	**0.008**
CA19‐9, U/mL	26.9 (0.1‐2259)	34.2 (0.7‐1690)	26.8 (0.2‐461)	0.282
TNM classification (UICC 8th) T factor (T1/T2/T3/T4)	8/10/1/11	10/21/5/23	9/17/3/12	0.857
TNM classification (UICC 8th) N factor (N0/N1/N2)	27/3/0	48/10/1	37/3/1	0.579
TNM classification (UICC 8th) M factor (M0/M1)	30/0	57/2	40/1	0.797
Resectability, R : BR : UR	17/6/7	22/21/16	22/13/6	0.243
**Upfront surgery/ NAC/ NCRT**	**9/0/21**	**2/2/55**	8/0/33	**0.002**
Albumin, mg/dL	3.8 (1.9‐4.5)	3.8 (2.5‐4.7)	3.8 (2.8‐4.6)	0.387
White blood cell counts	4700 (2670‐8130)	4610 (2470‐8480)	4960 (2950‐11 510)	0.369
Neutrophil	3210 (1050‐6830)	3000 (1360‐7930)	3010 (1750‐10 430)	0.948
Lymphocyte	1110 (290‐2220)	990 (270‐3260)	920 (270‐3030)	0.525
Hemoglobin	11.5 (8.7‐14.1)	11.8 (8.7‐15.0)	11.8 (8.1‐15.8)	0.494
Platelet counts, x 1000 /µL	203 (119‐340)	206 (60.0‐430)	220 (98.0‐370)	0.485
Spleen volume, mL	115 (41.8‐419)	114 (28.8‐277)	105 (21.8‐245)	0.934
Operative procedures (PD/SSPPD)	0/30	5/54	2/39	0.275
Operative duration (min)	535 (345‐818)	542 (351‐780)	537 (392‐793)	0.722
**Blood loss (mL)**	**547 (70‐2500)**	**160 (110‐5089)**	**523 (60‐1720)**	**< 0.001**
**LGV division, yes/no (yes%)**	**18/12 (60.0%)**	**53/6 (89.8%)**	36/5 (87.8%)	**0.001**
**LGV division/LGV‐PV/LGV‐SV**	**18/8/4**	**53/2/4**	3/2/36	**0.005**
**IMV division, yes/no (yes%)**	**10/20 (33.3%)**	35/24 (59.3%)	27/14 (65.9%)	**0.017**
**IMV division/ IMV‐SV/ IMV‐SMV**	**10/17/3**	35/24/0	27/13/1	**0.012**
C‐D >/= IIIa, yes/no (yes%)	4/26 (13.3%)	16/43 (27.1%)	5/36 (12.2%)	0.114
Pancreatic fistula (Grade B or C), yes/no (yes%)	0/30 (0.0%)	2/57 (3.4%)	1/40 (2.4%)	0.797
pPV positive, yes/no (yes%)	3/27 (10.0%)	16/43 (27.1%)	6/35 (14.6%)	0.102
R0 resection, yes/no (yes%)	24/6 (80.0%)	52/7 (88.1%)	38/3 (92.7%)	0.272
**Postoperative hospital stays, days**	**26 (15‐60)**	**38 (16‐118)**	**23 (14‐53)**	**< 0.001**

Abbreviations: BMI, body mass index; BR, borderline resectable; CA19‐9, carbohydrate antigen; C‐D, Clavien‐Dindo; CEA, carcinoembryonic antigen; IMV, inferior mesenteric vein; LGV, left gastric vein; NAC, neoadjuvant chemotherapy; NCRT, neoadjuvant chemoradiotherapy; PD, pancreaticoduodenectomy; pPV, pathological portal vein; R, resectable; R0 resection, curative resection; SAL, splenic artery ligation; SSPPD, subtotal stomach preserving PD; SV, splenic vein; SVP, splenic vein preservation; SVR, splenic vein resection; UICC, R, resectable; UR, unresectable.

### 3.2 Arterial blood supply to left‐sided area after PD‐SAL

**FIGURE 2:**
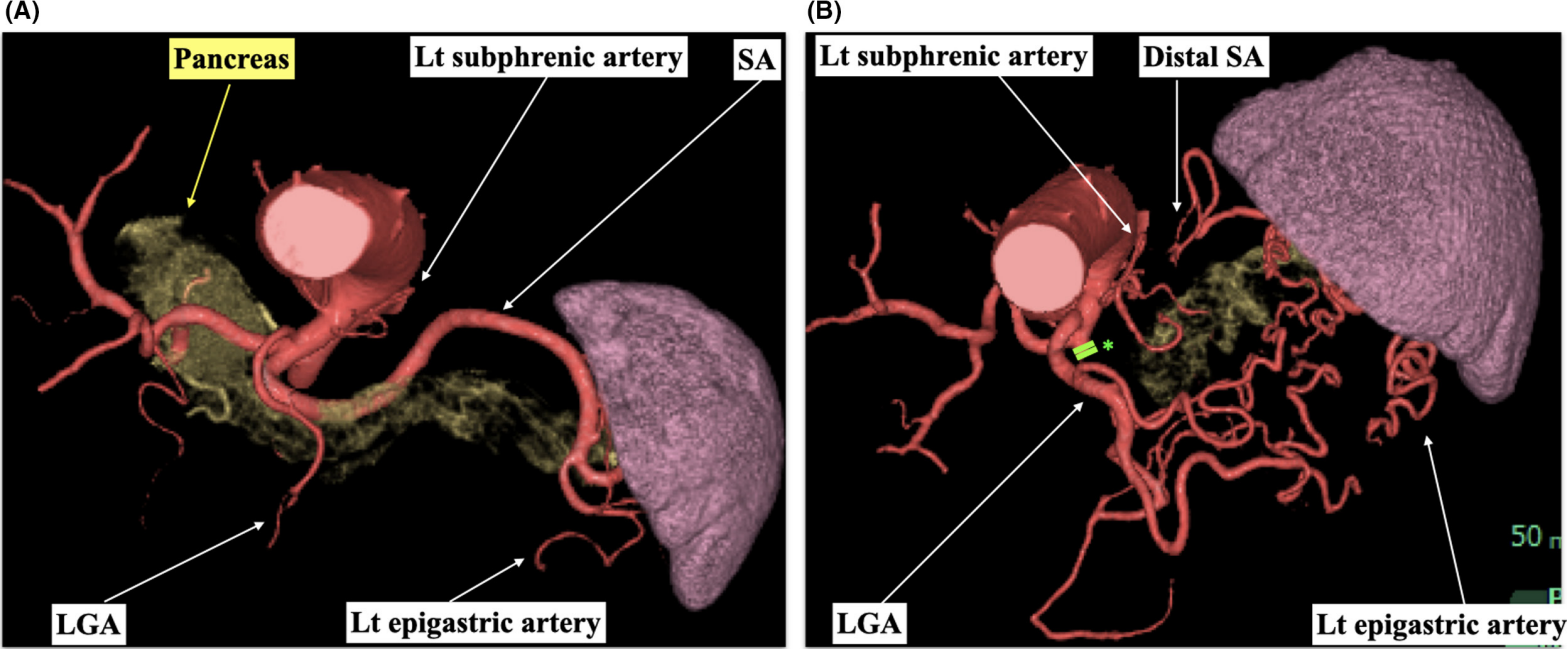
Arterial blood supply to the spleen. (A) Before PD‐SAL. (B) 12 mo after PD‐SAL. Arterial blood inflow to the spleen was derived from the enlarged LGA and left subphrenic artery through perigastric routes. SAL: splenic artery ligation, SA: splenic artery, LGA: left gastric artery, *clips

Blood supply to the spleen after PD‐SAL was mainly through the left gastric artery (LGA) and subphrenic artery, which passed through and around the stomach, and joined the distal SA and the spleen (Figure 2). On the dynamic CT image acquired within 14 d of PD‐SAL, blood supply from the LGA and subphrenic artery were identified in 100% (41/41) and 90.2% (37/41), respectively. There were no severe complications caused by SAL, including spleen necrosis and abscess. Partial splenic infarction was identified in 4.76% (2/41) in the PD‐SAL group; the infarct spleen volume occupied 17.0% and 8.9% of the total spleen volume, respectively. They developed no symptoms and required no additional treatments.

### 3.3 Postoperative variceal formation, development of collateral routes from the SV, and variceal bleeding due to LSPH

The incidence of variceal formation was significantly higher in the PD‐SVR group at postoperative 3 and 6 mo (PD‐SVP vs PD‐SVR vs PD‐SAL: 4.8% vs 53.2% vs 46.4%, *P* = .001 and 13.0% vs 71.2% vs 62.5%, P < .001), and was significantly higher in the PD‐SVR and PD‐SAL groups at postoperative 12 mo (PD‐SVP vs ‐SVR vs ‐SAL: 25.0% vs 87.5% vs 87.1%, P < .001) (Table 2). The rates of variceal formation in the PD‐SVP group were significantly lower among the three groups at each time. Each varix tended to develop faster in PD‐SVR compared with in PD‐SAL at each time, except for pancreatic varices at 12 mo; there were no significant differences.

**TABLE 2:** Postoperative development of varices and collateral routes at postoperative 3, 6 and 12 months

Postoperative LPH variables	PD‐SVP	PD‐SVR	PD‐SAL	*P*
Postoperative 3 months (+‐1mo)	n = 21	n = 47	n = 28	
**Postoperative variceal formation, yes/no (yes%)**	**1/20 (4.8%)**	**25/22 (53.2%)**	13 /15 (46.4%)	**0.001**
**Esophageal varices**	**0/21 (0.0%)**	14/33 (29.8%)	7/21 (25.0%)	**0.021**
**Gastric varices**	0/21 (0.0%)	**7/40 (14.9%)**	0/28 (0.0%)	**0.018**
Pancreatic varices	0/21 (0.0%)	2/45 (4.3%)	1/27 (3.6%)	1.000
**Colonic varices**	**1/20 (4.8%)**	**15/32 (31.9%)**	6/22 (21.4%)	**0.047**
**The development of collateral routes, yes/no (yes%)**	**2/19 (9.5%)**	**24/23 (51.1%)**	14/14 (50.0%)	**0.001**
Superior route	1/20 (4.8%)	1/46 (2.1%)	0/28 (0.0%)	0.512
**Inferior route**	**1/20 (4.8%)**	21/26 (44.7%)	12/16 (42.9%)	**0.004**
Spleno‐renal shunt	0/21 (0.0%)	0/47 (0.0%)	1/27 (3.6%)	0.510
Gastro‐renal shunt	1/20 (4.8%)	3/44 (6.4%)	2/26 (7.1%)	1.000
Postoperative 6 months (+‐1mo)	n = 23	n = 52	n = 40	
**Postoperative variceal formation, yes/no (yes%)**	**3/20 (13.0%)**	**37/15 (71.2%)**	**25/15 (62.5%)**	**< 0.001**
**Esophageal varices**	**0/23 (0.0%)**	**19/33 (36.5%)**	10/30 (25.0%)	**0.004**
**Gastric varices**	1/22 (4.3%)	14/38 (26.9%)	7/33 (17.5%)	**0.069**
**Pancreatic varices**	1/22 (4.3%)	7/45 (13.5%)	4/36 (10.0%)	0.572
**Colonic varices**	**1/22 (4.3%)**	**25/27 (48.1%)**	17/23 (42.5%)	**0.001**
**The development of collateral routes, yes/no (yes%)**	**2/21 (8.7%)**	**35/17 (67.3%)**	25/15 (62.5%)	**< 0.001**
Superior route	1/22 (4.3%)	0/52 (0.0%)	1/39 (2.5%)	0.293
**Inferior route**	**1/22 (4.3%)**	**30/22 (57.7%)**	22/18 (55.0%)	**< 0.001**
Spleno‐renal shunt	0/23 (0.0%)	2/50 (3.8%)	1/39 (2.5%)	0.628
Gastro‐renal shunt	0/23 (0.0%)	4/48 (7.7%)	2/38 (5.0%)	0.384
Postoperative 12 months (+‐2mo)	n = 24	n = 40	n = 31	
**Postoperative variceal formation, yes/no (yes%)**	**6/18 (25.0%)**	**35/5 (87.5%)**	**27/4 (87.1%)**	**< 0.001**
**Esophageal varices**	**2/22 (8.3%)**	**20/20 (50.0%)**	10/21 (32.3%)	**0.003**
**Gastric varices**	1/23 (4.2%)	**15/25 (37.5%)**	9/22 (29.0%)	**0.012**
**Pancreatic varices**	**2/22 (8.3%)**	12/28 (30.0%)	**13/18 (41.9%)**	**0.022**
**Colonic varices**	**1/23 (4.2%)**	**26/14 (65.0%)**	17/14 (54.8%)	**< 0.001**
**The development of collateral routes, yes/no (yes%)**	**2/22 (8.3%)**	**30/10 (75.0%)**	22/9 (71.0%)	< 0.001
Superior route	1/23 (4.2%)	0/40 (0.0%)	1/30 (3.2%)	0.333
**Inferior route**	**1/23 (4.2%)**	**25/15 (62.5%)**	**21/10 (67.7%)**	**< 0.001**
Spleno‐renal shunt	0/24 (0.0%)	2/38 (5.0%)	1/30 (3.2%)	0.541
Gastro‐renal shunt	1/23 (4.2%)	4/36 (10.0%)	1/30 (3.2%)	0.448

n: total number of patients who underwent enhanced CT at each time.

Abbreviations: PD, pancreaticoduodenectomy; SVP, splenic vein preservation; SVR, splenic vein resection; SAL, splenic artery ligation.

The development rates of collateral routes were significantly lower in the PD‐SVP group and significantly higher in the PD‐SVR group (Table 2). The rates in PD‐SAL were similar to those in PD‐SVR: 51.1% vs 50.0% at 3 mo, 67.3% vs 62.5% at 6 mo, and 75.0% vs 71.0% at 12 mo. The majority of the collateral route developed was the inferior route, which quickly developed in PD‐SVR and PD‐SAL as opposed to PD‐SVP. There were no significant differences in the development of the superior route and spleno‐systemic shunts such as spleno‐renal and gastro‐renal shunts.

Variceal bleeding was identified in four patients who underwent PD‐SVR. One patient developed bleeding in the ascending jejunal limb from rupture of pancreatic varices at 6 mo after PD‐SVR and underwent emergency splenectomy with preservation of the LGV, which was open on enhanced CT. Another patient developed gastrointestinal bleeding from the rupture of gastric varices at 6 mo after PD‐SVR. She succumbed to disseminated intravascular coagulation, although endoscopic homeostasis, transarterial embolization, and distal gastrectomy were performed. However, another patient developed hemorrhage from esophageal varices at 18 mo after PD‐SVR and was treated with endoscopic variceal ligation. Another patient developed rectal bleeding from colonic varices and underwent colectomy at 98 mo after PD‐SVR.

### 3.4 Perioperative risk factors for variceal formation at postoperative 3, 6, and 12 mo

Multivariate analyses identified large tumor size on CT (odds ratio [OR], 1.07; *P* = .041), high albumin level (OR, 10.6; *P* < .001), SVR (OR, 25.9; *P* = .004), and SAL (OR, 19.6; *P* = .009) were risk factors for variceal formation at postoperative 3 mo (Table S1). Albumin level (OR, 4.67; *P* = .007), SVR (OR, 17.0; *P* < .001), and SAL (OR, 12.4; *P* < .001) compared with SVP were identified as risk factors of variceal formation at postoperative 6 mo (Table S2). SVR (OR, 21.0; *P*  < .001) and SAL (OR, 20.3; *P* < .001) compared with SVP were identified as risk factors for variceal formation at postoperative 12 mo (Table S3).

### 3.5 Platelet count and spleen volume

**FIGURE 3:**
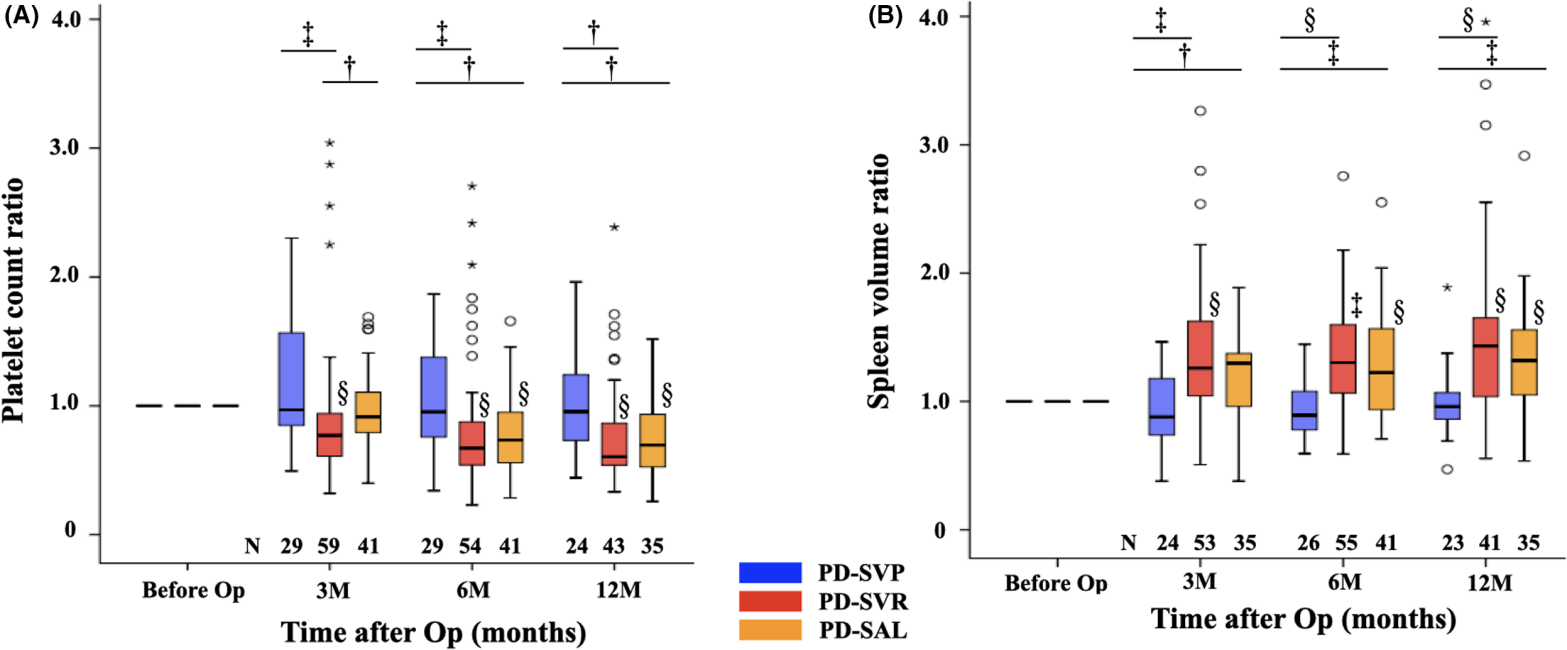
Serial changes in the platelet count and spleen volume ratios. Platelet count and spleen volume ratios in PD‐SVP were maintained postoperatively. The platelet count ratio significantly decreased from 3 mo in PD‐SVR and from 6 mo in PD‐SAL. Among the three groups, the platelet count ratio was significantly lower in PD‐SVR at 3 mo, and in PD‐SVR and PD‐SAL at 6 and 12 mo, respectively. The spleen volume ratio significantly decreased from 3 mo in PD‐SVR and 6 mo in PD‐SAL. Among the three groups, the spleen volume ratio was significantly higher in PD‐SVR and PD‐SAL at 3, 6, and 12 mo. M, month; N, number; o, outlier, Op, operation; PD, pancreaticoduodenectomy; SAL, splenic artery ligation; SVP, splenic vein preservation; SVR, splenic vein resection, *extreme outlier (three times or more), ^†^ *P* < .05, ^‡^ *P* < .01, ^§^ *P* < .001

Figure 3 shows serial changes in platelet count and spleen volume ratios at postoperative 3, 6, and 12 mo. The platelet count ratio in the PD‐SVR group significantly decreased at 3, 6, and 12 mo compared with the preoperative value: 0.77 (0.32–3.04), 0.67 (0.23–2.70), and 0.60 (0.33–2.39), *P* < .001, respectively; that in the ratio in the PD‐SAL group was 0.91 (0.40–1.69) at 3 mo (comparable to the preoperative value), but significantly decreased at 6 and 12 mo compared with the preoperative value: 0.73 (0.23–1.66) and 0.69 (0.26–1.52), respectively (*P* < .001), and that in the ratio in the PD‐SVP group was maintained without any postoperative significant change. The ratio in PD‐SVR at 3 mo was significantly lower than those in PD‐SVP and PD‐SAL (*P* = .001 and *P* = .46, respectively), and significantly lower than that in PD‐SVP at 6 and 12 mo (*P* = .003 and *P* = .012, respectively). The ratio in PD‐SAL was comparable to that in PD‐SVP at 3 mo but significantly lower at 6 and 12 mo (*P* = .011 and *P* = .012, respectively). In PD‐SVP, SVR, and SAL, thrombocytopenia of grade 2 or higher (<75,000/mL) occurred in none at 3 mo, 3.4% (1/29) vs 7.4% (4/54) vs 2.4% (1/41) at 6 mo, and 0% (0/24) vs 4.7% (2/43) vs 2.9% (1/35) at 12 mo after surgery. At postoperative 6 and 12 mo, the rate of thrombocytopenia of grade 2 or higher tended to be higher in PD‐SVR, although there were no significant differences.

The spleen volume ratio in PD‐SVR significantly increased at 3, 6, and 12 mo, compared with the preoperative value: 1.24 (0.56–3.26), *P < .001*; 1.34 (0.59–2.76), *P* = .003; and 1.42 (0.56–3.96), *P < .001*. The ratio in PD‐SAL was 1.31 (0.38–1.78) at 3 mo (comparable to the preoperative value), but significantly increased at 6 and 12 mo compared with the preoperative value: 1.32 (0.71–2.04), *P*  < .001 and 1.34 (0.54–2.92), *P* < .001. The ratio in PD‐SVP was maintained at 3, 6, and 12 mo. Among the three groups, the spleen volume ratio in PD‐SVR was significantly higher than that in PD‐SVP at 3, 6, and 12 mo (*P* = .001, *P* < .001, and *P* < .001, respectively). The ratio in PD‐SAL was also higher than that in PD‐SVP at 3, 6, and 12 mo (*P* = .011, *P* = .001, and *P* = .006, respectively).

### 3.6 Variceal formation at 12 mo postoperative according to venous drainage routes from the divided SV

The LGV and IMV were preserved more frequently in PD‐SVP (Table 1). Collateral routes rarely developed in PD‐SVP (Table 2). Therefore, the effect of venous drainage routes in LSPH was evaluated by focusing on the patients with SV division (PD‐SVR and PD‐SAL).

The preservation of the LGV‐PV confluence was identified in three patients and was associated with slower development of colonic varices and inferior routes and faster development of superior routes. Gastro‐renal shunts quickly developed in patients with the LGV‐SV confluence preservation. Between LGV‐SV preservation and LGV division, there was no significant difference in the rates of variceal formation and collateral development: 100% (5/5) vs 87.3% (55/63) and 80% (4/5) vs 74.6% (47/63) (Table 3). The preservation of the IMV‐SMV confluence was only identified in one patient who did not develop varices or collateral routes. Between the IMV‐SMV preservation and the IMV division, there was no significant difference in the rates of variceal formation and collateral development: 92.3% (24/26) vs 86.4% (38/44) and 76.9% (20/26) vs 72.7% (32/44) (Table 4).

**TABLE 3:** The development of varices and collateral routes at postoperative 12 months according to the variance of the LGV confluence in the patients with the SV division

	LGV preservation (n = 8)		LGV division (n = 63)	*P*
	LGV‐PV (n = 3)	LGV‐SV (n = 5)		
PD‐SVR/SAL	1/2	3/2	36/27	0.708
Postoperative variceal formation, yes/no (yes%)	2/1 (66.7%)	5/0 (100.0%)	55/8 (87.3%)	0.390
Esophageal varices	2/1 (66.7%)	3/2 (60.0%)	25/38 (39.7%)	0.461
Gastric varices	2/1 (66.7%)	3/2 (60.0%)	20/43 (31.7%)	0.438
Pancreatic varices	0/3 (0.0%)	4/1 (80.0%)	39/24 (61.9%)	0.306
Colonic varices	1/9 (10.0%)	4/5 (44.4%)	39/37 (51.3%)	0.066
The development of collateral routes, yes/no (yes%)	1/2 (33.3%)	4/1 (80.0%)	47/16 (74.6%)	0.271
**Superior spleno‐portal route**	**1/2 (33.3%)**	0/5 (0.00%)	**0/63 (0.0%)**	**< 0.001**
**Inferior spleno‐portal route**	**0/3 (0.0%)**	2/3 (40.0%)	**44/19 (69.8%)**	**0.023**
Spleno‐renal route	0/3 (0.0%)	0/5 (0.00%)	3/60 (4.8%)	0.820
**Gastro‐renal route**	0/3 (0.0%)	**3/2 (60.0%)**	**2/61 (3.2%)**	**< 0.001**

Abbreviations: LGV, left gastric vein; PD, pancreaticoduodenectomy; SAL, splenic artery ligation; SV, splenic vein; SVP, splenic vein preservation; SVR, splenic vein resection.

**TABLE 4:** The development of varices and collateral routes at postoperative 12 months according to the variance of IMV confluence in the patients with the SV division

	IMV preservation (n = 27)		IMV division (n = 44)	*P*
	IMV‐SV (n = 26)	IMV‐PV (n = 1)		
PD‐SVP/SVR/SAL	15/11	0/1	25/19	0.518
**Postoperative variceal formation, yes/no (yes%)**	24/2 (92.3%)	**0/1 (0.0%)**	38/6 (86.4%)	**0.023**
Esophageal varices	9/17 (34.6%)	0/1 (0.0%)	21/23 (47.7%)	0.388
Gastric varices	8/18 (30.8%)	0/1 (0.0%)	16/28 (36.4%)	0.688
Pancreatic varices	8/18 (30.8%)	0/1 (0.0%)	17/27 (38.6%)	0.608
Colonic varices	18/8 (69.2%)	0/1 (0.0%)	25/19 (56.8%)	0.271
The development of collateral routes, yes/no (yes%)	20/6 (76.9%)	0/1 (0.0%)	32/12 (72.7%)	0.232
Superior spleno‐portal route	0/26 (0.0%)	0/1 (0.0%)	1/43 (2.3%)	0.733
Inferior spleno‐portal route	19/7 (73.1%)	0/1 (0.0%)	27/17 (61.4%)	0.241
Spleno‐renal route	1/25 (3.8%)	0/1 (0.0%)	2/42 (4.5%)	0.968
Gastro‐renal route	2/24 (7.5%)	0/1 (0.0%)	3/41 (6.8%)	0.953

Abbreviations: IMV, inferior mesenteric vein; PD, pancreaticoduodenectomy; SAL, splenic artery ligation; SV, splenic vein; SVP, splenic vein preservation; SVR, splenic vein resection.

Among the postoperative developed collateral routes, the most frequently identified route was the inferior route: 83.3% (25/30) in SVR and 95.5% (21/22) in SAL at 12 mo postoperative (Table 2). The incidence of variceal formation was 95.7% (44/46) in patients with inferior collateral routes development and 72.0% (18/25) in those without, showing a significant difference (*P* = .007) (Table 5). The incidence of pancreatic and colonic varices was significantly higher in the patients with inferior route development than in those without: 43.5% vs 20.0%, *P* = .048 and 76.1% vs 32.0%, P < .001.

**TABLE 5:** The relationship between variceal formation and an inferior collateral route developing at postoperative 12 months in the patients with the SV division

	The development of an inferior collateral route		*P*
	No (n = 25)	Yes (n = 46)	
PD‐SVR/SAL	15/10	21/25	0.708
**Postoperative variceal formation, yes/no (yes%)**	**18/7 (72.0%)**	**44/2 (95.7%)**	**0.007**
Esophageal varices	10/15 (40.0%)	20/26 (43.5%)	0.777
Gastric varices	8/17 (32.0%)	16/30 (34.8%)	0.813
**Pancreatic varices**	**5/20 (20.0%)**	**20/26 (43.5%)**	**0.048**
**Colonic varices**	**8/17 (32.0%)**	**35/11 (76.1%)**	**< 0.001**

Abbreviations: PD, pancreaticoduodenectomy; SAL, splenic artery ligation; SV, splenic vein; SVR, splenic vein resection.

## 4 DISCUSSION

In this study, we investigated the efficacy of splenic artery (SA) ligation in preventing LSPH and elucidated the following: (a) SAL delayed the occurrence of LSPH but could not prevent it in terms of digestive variceal formation, thrombocytopenia, and splenomegaly; (b) Variceal hemorrhage within 12 mo occurred only in PD‐SVR but did not occur in PD‐SAL; (c) The arterial supply to the spleen after SAL was mainly from the left gastric (100%) and subphrenic (93%) arteries through or around the stomach without causing any major complications; and (d) The preservation of LGV‐SV or IMV‐SV confluence had no effect in preventing variceal formation.

In PD with the PV/SMV confluence resection, the divided SV is rarely reconstructed. In a study, the SV was not reconstructed in more than 95% of the 251 patients who underwent PD with SV resection between 2005 and 2014.^1^ Most surgeons pay no attention to LSPH after SV division because they believe venous blood from the divided SV will successfully drain to the venous circulation of the stomach and colon without causing any major problems. However, the accumulated blood in the spleen due to SV division causes LSPH, which then manifests as variceal formation, collateral development, thrombocytopenia, and splenomegaly.^1^, ^2^ Particularly, variceal formation represented by esophageal, gastric, pancreatic, and colonic varices has a risk of lethal hemorrhage requiring emergency treatment.^1^, ^2^, ^3^ LSPH is a major problem that needs to be addressed.

Preservation of the LGV or IMV prevents postoperative LSPH in a small number of patients.^5^, ^6^, ^18^, ^19^ In this study, however, the preservation of the LGV‐SV or IMV‐SV confluence had no effect on the occurrence of LSPH. The accumulated blood in the spleen due to SV division, flowed to the perigastric circulation via the preserved LGV (resulting in the development of esophageal and gastric varicose routes) or to the colonic marginal vein via the preserved IMV and omental arcade forming inferior routes.^18^, ^20^ In this study the development of inferior routes was associated with variceal formation, including pancreatic and colonic varices. This result suggests that LSPH developed with inferior routes but also with varicose routes if the drainage veins to portal circulation such as the IMV‐SMV confluence, MCV and superior right colic vein (SRCV) were not preserved. In contrast, the preservation of the LGV‐PV or IMV‐PV theoretically secures the venous return to the portal circulation from the divided SV. LGV‐PV preservation promotes superior routes formation from the divided SV, contributing to the suppression of colonic variceal formation and inferior routes development. IMV‐SMV preservation did not cause varicose routes. However, it is difficult to preserve such venous drainage routes to the PV. First, the presence of LGV‐PV or IMV‐SMV is determined by innate venous variation. Second, they are commonly sacrificed to allow oncologic clearance and PV/SMV mobility. If preserved, the caliber size and flow velocity of such veins might be insufficient in preventing LSPH. We observed pancreatic variceal hemorrhage at 6 mo postoperative even though the LGV was preserved and the patency was confirmed on enhanced CT. Preservation of two or more of the LGV, MCV, and SRCV arcade is critical in preventing LSPH^7^; in their analysis of 88 PD patients with SV ligation or occlusion, LSPH developed in all 29 (100%) patients in whom none of the three critical veins were preserved, and in 12 (24%) of the 51 patients with only one of the critical veins preserved. In contrast, LSPH developed in none (0%) of eight patients in whom two or three of the critical veins were preserved. Therefore, they recommended the outflow reconstruction of the SV when two or more of all three potential collateral veins of LGV, MCV, and SRCV are divided.^7^ Anastomosis between the SV and left renal vein is an anatomically valid procedure. Although the bypass was performed (by experienced hands) without increasing operative duration and complications, the postoperative patency was 55%; thus, further refinement is necessary.

We previously reported the efficacy of PD‐SAR in preventing LSPH.^2^ Variceal formation in PD‐SAR was significantly lower than that in PD‐SVR within 6 mo postoperatively. SAR could reduce the portal venous pressure and portal modulations such as SA embolization, ligation, or splenectomy, which have been performed to reduce portal hyperperfusion and hypertension, contributing to successful liver regeneration in the living‐donor liver transplant using a small graft.^9^, ^10^, ^21^ For preventing LSPH, concomitant SAL has been performed in PD with PV/SMV resection and SV division since September 2016.

Splenic artery ligation is a simple procedure of just clumping the root of the SA without requiring complex techniques and additional operative duration. Furthermore, SAL is safe and does not cause major complications such as severe splenic infarction, necrosis, and abscess, which require antibiotic treatment, drainage, or splenectomy. The blood supply to the spleen was secured from LGA and left subphrenic artery through the stomach. Arterial inflow into the spleen would relatively decrease in the short term after SAL, but it would gradually develop from the enlarged LGA and subphrenic artery. As a result, the rate of variceal formation was slightly lower in SAL than in SVR at postoperative 3 and 6 mo, but this slight superiority disappeared at 12 mo. The protective effect of SAL (from thrombocytopenia and splenomegaly) was also identified at 3 mo, but this effect disappeared after 6 mo. Even in the multivariate analysis of risk factors for variceal formation, SVR was a strong risk factor and SAL was not protective enough to eliminate the significant difference. However, no variceal hemorrhage was identified within 12 mo after PD‐SAL, although 2 (3.39%) of the 59 patients developed variceal hemorrhage within 6 mo of PD‐SVR. This result might suggest that SAL controlled LSPH for not causing variceal hemorrhage, although varices developed. Yamada et al reported SAL was effective in preventing LSPH in patients undergoing PD with PV/SMV resection.^12^ In 63 patients with SV division, SAL significantly decreased the rate of variceal formation compared to patients without SAL at around 12 mo postoperative: 14.3% vs 47.6%, *P* = .005. However, they preserved the MCV in more than half of the patients, while we resected it in all patients for lymph node dissection by the anterior approach to the SMA. The preserved MCV can become a significant drainage route to the SMV in inferior routes. Surgical differences between this study and theirs might have influenced the effect of SAL on the development of LSPH.

The limitation of this study was that it was a relatively short‐term, retrospective, and single‐center study. LSPH could be precisely evaluated over time in more than 100 patients who underwent unified perioperative treatments in one institution. However, the results of this study should be validated in a prospective multicenter clinical study evaluating the clinical impact of SAL on LSPH in the long term. Large tumor size and high albumin level were identified as risk factors for postoperative variceal formation at 3 and 6 mo, respectively. A large tumor generally demands extensive resection beyond the pancreas. As a result, venous drainage routes from the SV might decrease and varicose routes might develop. High albumin levels reflect good patients' status, but may promote the development of varicose routes.

In conclusion, although SAL could not prevent LSPH, it could delay the occurrence of LSPH without causing variceal hemorrhage after PD with PV/SMV confluence resection.

## DISCLOSURE

Ethical Approval: Approval of the research protocol: The study protocol was approved by the Medical Ethics Committee of Mie University Hospital.

Informed Consent: Informed consent was obtained from each participant on an opt‐out basis.

Registry and the Registration No. of the study/Trial: No. H2019‐070/IRB in Mie University Hospital.

Animal Studies: N/A.

Conflict of Interest: The authors declare no conflicts of interest for this article.

## Supporting information

Table S1

Table S2

Table S3
