# Supplementary material for: Concomitant splenic artery ligation has no preventive effect on left‐sided portal hypertension following pancreaticoduodenectomy with the resection of the portal and superior mesenteric vein confluence for pancreatic ductal adenocarcinoma
Source: Ann Gastroenterol Surg. 2022 Feb 10;6(3):420–9. doi: 10.1002/ags3.12545 (PMC9130910; doi:10.1002/ags3.12545)
Supplement: Supplementary file 1 — Table S1 [file AGS3-6-420-s001.docx]

**Supplemental Table 1. Peri-operative factors contributing to varices formation at postoperative 3 months by uni- and multivariate analyses**

| **Perioperative variables** | **Varices formation No**  **n=57** | **Varices formation Yes**  **n=39** | **P** | **Odd’s ratio** | **95% CI** | **p** |
| --- | --- | --- | --- | --- | --- | --- |
| Age | 67 (50-83) | 65 (41-85) | 0.147 |  |  |  |
| Male/Female | 31/26 | 25/14 | 0.343 |  |  |  |
| BMI, kg/m2 | 21.3 (15.2-27.1) | 20.4 (14.0–27.4) | 0.152 |  |  |  |
| **Maximum tumor size on CT, mm** | **22.7 (11.2 -45.9)** | **26.1 (12.0-44.9)** | **0.021** | **1.07** | **1.003-1.138** | **0.041** |
| Performance status 0/1/2/3 | 44/11/2/0 | 25/13/0/1 | 0.111 |  |  |  |
| TNM classification (UICC 8th) T factor (T1/T2/T3/T4) | 15/17/4/21 | 3/18/4/14 | 0.099 |  |  |  |
| TNM classification (UICC 8th) N factor (N0/N1/N2) | 52/5/0 | 36/3/0 | 0.582 |  |  |  |
| Resectability, R : BR : UR | 27/18/12 | 17/10/12 | 0.542 |  |  |  |
| Upfront surgery/ NAC/ NCRT | 8/2/47 | 3/0/36 | 0.378 |  |  |  |
| **Albumin, mg/dL** | **3.7 (1.9-4.6)** | **4.0 (2.8-4.7)** | **< 0.001** | **10.6** | **2.66-42.43** | **< 0.001** |
| White blood cell counts | 4,820 (2,720-8,840) | 4,880 (2,670-8,160) | 0.541 |  |  |  |
| Hemoglobin | 11.6 (8.1-14.7) | 11.8 (9.0-15.8) | 0.085 |  |  |  |
| Platelet counts, x 1000 /uL | 207 (75.0-430) | 220 (84.0-423) | 0.279 |  |  |  |
| Spleen volume, ml | 118 (36.4- 419) | 104 (21.8-225) | 0.890 |  |  |  |
| Operative procedures (PD/SSPPD) | 5/52 | 2/37 | 0.401 |  |  |  |
| **Management of SV and SA** |  |  | **0.001** |  |  | **0.014** |
| **SVP** | **20** | **1** |  | 1 | - | - |
| **SVR** | **22** | **25** |  | **25.9** | **2.90-230.4** | **0.004** |
| **SAL** | 15 | 13 |  | **19.6** | **2.09-183.9** | **0.009** |
| Operative duration (min) | 540 (345-818) | 550 (371-793) | 0.602 |  |  |  |
| Blood loss (ml) | 825 (60 - 4,930 ) | 735 (110-4,739) | 0.806 |  |  |  |
| LGV division, yes/no (yes %) | 48/9 (84.2 %) | 33/6 (84.6 %) | 0.957 |  |  |  |
| LGV division/LGV-PV/LGV-SV | 48/5/4 | 33/3/3 | 1.000 |  |  |  |
| IMV division, yes/no (yes %) | 36/21 (63.2 %) | 24/15 (61.5 %) | 0.872 |  |  |  |
| IMV division/ IMV-SV/ IMV-SMV | 36/19/2 | 24/15/0 | 0.64 |  |  |  |
| C-D >/= IIIa, yes/no (yes %) | 13/44 (22.8 %) | 8/31 (20.5 %) | 0.789 |  |  |  |
| Pancreatic fistula (Grade B or C), yes/no (yes %) | 2/55 (3.5 %) | 0/39 (0.0 %) | 0.350 |  |  |  |
| pPV positive, yes/no (yes%) | 9/48 (15.8 %) | 9/30 (23.1 %) | 0.369 |  |  |  |
| R0 resection, yes/no (yes %) | 51/6 (89.5 %) | 35/4 (89.7 %) | 0.622 |  |  |  |
| Postoperative hospital stays, days | 30 (16-88) | 35 (14-118) | 0.305 |  |  |  |

SVP: splenic vein preservation, SVR: splenic vein resection, SAL: splenic artery ligation, BMI: body mass index, UICC, R: resectable, BR: borderline resectable, UR: unresectable, NAC: neoadjuvant chemotherapy, NCRT: neoadjuvant chemoradiotherapy, PD: pancreaticoduodenectomy, SSPPD: subtotal stomach preserving PD, LGV: left gastric vein, IMV: inferior mesenteric vein, SV: splenic vein, C-D: Clavien-Dindo, pPV: pathological portal vein, R0 resection: curative resection
