# Supplementary material for: Concomitant splenic artery ligation has no preventive effect on left‐sided portal hypertension following pancreaticoduodenectomy with the resection of the portal and superior mesenteric vein confluence for pancreatic ductal adenocarcinoma
Source: Ann Gastroenterol Surg. 2022 Feb 10;6(3):420–9. doi: 10.1002/ags3.12545 (PMC9130910; doi:10.1002/ags3.12545)
Supplement: Supplementary file 2 — Table S2 [file AGS3-6-420-s003.docx]

**Supplemental Table 2. Peri-operative factors contributing to varices formation at postoperative 6 months by uni- and multivariate analyses**

| **Perioperative variables** | **Varices formation No**  **n=50** | **Varices formation Yes**  **n=65** | **P** | | | **Odd’s ratio** | | **95% CI** | | **p** |
| --- | --- | --- | --- | --- | --- | --- | --- | --- | --- | --- |
| Age | 68 (50-83) | 67 (41-85) | | 0.130 |  | |  | |  | |
| Male/Female | 25/25 | 40/25 | | 0.216 |  | |  | |  | |
| BMI, kg/m2 | 21.3 (15.9-28.3) | 20.6 (14.0–27.9) | | 0.408 |  | |  | |  | |
| Maximum tumor size on CT, mm | 21.9 (11.2 -45.9) | 25.2 (10.6-44.9) | | 0.110 |  | |  | |  | |
| Performance status 0/1/2/3 | 34/14/2/0 | 43/19/2/1 | | 1.000 |  | |  | |  | |
| TNM classification (UICC 8th) T factor (T1/T2/T3/T4) | 15/13/4/18 | 9/31/4/21 | | 0.061 |  | |  | |  | |
| TNM classification (UICC 8th) N factor (N0/N1/N2) | 42/7/1 | 57/7/1 | | 0.888 |  | |  | |  | |
| Resectability, R : BR : UR | 23/18/9 | 31/20/14 | | 0.807 |  | |  | |  | |
| Upfront surgery/ NAC/ NCRT | 10/1/39 | 7/1/57 | | 0.373 |  | |  | |  | |
| **Albumin, mg/dL** | **3.8 (2.5-4.6)** | **3.9 (2.8-4.7)** | | **0.005** | **4.67** | | **1.53-14.3** | | **0.007** | |
| White blood cell counts | 4,790 (2,470-11,510) | 4,930 (2,670-8,160) | | 0.676 |  | |  | |  | |
| Hemoglobin | 11.4 (8.7-14.7) | 11.9 (8.1-15.8) | | 0.046 |  | |  | |  | |
| Platelet counts, x 1000 /uL | 205 (60.0-344) | 213 (84.0-430) | | 0.253 |  | |  | |  | |
| Spleen volume, ml | 121 (28.8- 419) | 107 (21.8-238) | | 0.446 |  | |  | |  | |
| Operative procedures (PD/SSPPD) | 3/47 | 2/63 | | 0.377 |  | |  | |  | |
| **Management of SV and SA** |  |  | | **< 0.001** |  | |  | | **< 0.001** | |
| **SVP** | **20** | **3** | |  | 1 | | - | | - | |
| **SVR** | **15** | **37** | |  | **17** | | **4.19-68.6** | | **< 0.001** | |
| **SAL** | 15 | 25 | |  | **12.4** | | **3.02-51.1** | | **< 0.001** | |
| Operative duration (min) | 536 (351-746) | 534 (345-793) | | 0.817 |  | |  | |  | |
| Blood loss (ml) | 762 (60 - 5,089 ) | 720 (110-4,930) | | 0.674 |  | |  | |  | |
| LGV division, yes/no (yes %) | 38/12 (76.0 %) | 55/10 (84.6 %) | | 0.244 |  | |  | |  | |
| LGV division/LGV-PV/LGV-SV | 38/9/3 | 55/4/6 | | 0.126 |  | |  | |  | |
| IMV division, yes/no (yes %) | 25/25 (50.0 %) | 40/25 (61.5 %) | | 0.216 |  | |  | |  | |
| IMV division/ IMV-SV/ IMV-SMV | 25/3/22 | 40/25/0 | | 0.092 |  | |  | |  | |
| C-D >/= IIIa, yes/no (yes %) | 11/39 (22.0 %) | 12/53 (18.5 %) | | 0.638 |  | |  | |  | |
| Pancreatic fistula (Grade B or C), yes/no (yes %) | 1/49 (2.0 %) | 1/64 (1.5 %) | | 0.683 |  | |  | |  | |
| pPV positive, yes/no (yes%) | 7/43 (14.0 %) | 13/52 (20.0 %) | | 0.400 |  | |  | |  | |
| R0 resection, yes/no (yes %) | 44/6 (88.0 %) | 59/6 (90.8 %) | | 0.630 |  | |  | |  | |
| Postoperative hospital stays, days | 27 (15-74) | 32 (14-118) | | 0.240 |  | |  | |  | |

SVP: splenic vein preservation, SVR: splenic vein resection, SAL: splenic artery ligation, BMI: body mass index, UICC, R: resectable, BR: borderline resectable, UR: unresectable, NAC: neoadjuvant chemotherapy, NCRT: neoadjuvant chemoradiotherapy, PD: pancreaticoduodenectomy, SSPPD: subtotal stomach preserving PD, LGV: left gastric vein, IMV: inferior mesenteric vein, SV: splenic vein, C-D: Clavien-Dindo, pPV: pathological portal vein, R0 resection: curative resection
