# Supplementary material for: Concomitant splenic artery ligation has no preventive effect on left‐sided portal hypertension following pancreaticoduodenectomy with the resection of the portal and superior mesenteric vein confluence for pancreatic ductal adenocarcinoma
Source: Ann Gastroenterol Surg. 2022 Feb 10;6(3):420–9. doi: 10.1002/ags3.12545 (PMC9130910; doi:10.1002/ags3.12545)
Supplement: Supplementary file 3 — Table S3 [file AGS3-6-420-s002.docx]

**Supplemental Table 3. Peri-operative factors contributing to varices formation at postoperative 12 months by uni- and multivariate analyses**

| **Perioperative variables** | **Varices formation No**  **n=27** | **Varices formation Yes**  **n=68** | **P** | **Odd’s ratio** | | **95% CI** | | **p** |
| --- | --- | --- | --- | --- | --- | --- | --- | --- |
| Age | 69 (51-83) | 66 (41-85) | 0.091 |  |  | |  | |
| Male/Female | 14/13 | 42/26 | 0.376 |  |  | |  | |
| BMI, kg/m2 | 20.5 (18.3-27.1) | 21.3 (14.0–27.9) | 0.246 |  |  | |  | |
| Maximum tumor size on CT, mm | **20.3 (11.2 -34.8)** | **23.6 (10.6-45.9)** | **0.011** |  |  | |  | |
| Performance status 0/1/2/3 | 20/7/0/0 | 46/17/4/1 | 0.751 |  |  | |  | |
| TNM classification (UICC 8th) T factor (T1/T2/T3/T4) | 10/5/1/11 | 13/28/3/24 | 0.104 |  |  | |  | |
| TNM classification (UICC 8th) N factor (N0/N1/N2) | 24/3/0 | 60/8/0 | 0.619 |  |  | |  | |
| Resectability, R : BR : UR | 15/7/5 | 33/21/14 | 0.822 |  |  | |  | |
| Upfront surgery/ NAC/ NCRT | **8/0/19** | **6/2/60** | **0.034** |  |  | |  | |
| Albumin, mg/dL | 3.8 (3.1-4.4) | 3.9 (2.8-4.7) | 0.148 |  |  | |  | |
| White blood cell counts | 4,690 (2,720-8,840) | 4,940 (2,470-11,510) | 0.882 |  |  | |  | |
| Hemoglobin | 11.4 (9.1-14.1) | 11.8 (8.1-15.8) | 0.145 |  |  | |  | |
| Platelet counts, x 1000 /uL | 217 (119-339) | 206 (75.0-430) | 0.961 |  |  | |  | |
| Spleen volume, ml | 121 (41.8- 419) | 113 (21.8-277) | 0.433 |  |  | |  | |
| Operative procedures (PD/SSPPD) | 0/27 | 4/64 | 0.256 |  |  | |  | |
| **Management of SV and SA** |  |  | **< 0.001** |  |  | | **< 0.001** | |
| **SVP** | **18** | 6 |  | 1 | - | | - | |
| **SVR** | **5** | **35** |  | **21** | **5.63-78.3** | | **< 0.001** | |
| **SAL** | **4** | **27** |  | **20.3** | **5.00-82.0** | | **< 0.001** | |
| Operative duration (min) | 532 (355-746) | 539 (345-793) | 0.710 |  |  | |  | |
| Blood loss (ml) | 630 (70 - 2,784) | 641 (60-4,930) | 0.615 |  |  | |  | |
| LGV division, yes/no (yes %) | 19/8 (70.4 %) | 57/11 (83.8 %) | 0.139 |  |  | |  | |
| LGV division/LGV-PV/LGV-SV | 19/6/2 | 57/4/7 | 0.075 |  |  | |  | |
| IMV division, yes/no (yes %) | 11/16 (40.7 %) | 40/28 (58.8 %) | 0.111 |  |  | |  | |
| IMV division/ IMV-SV/ IMV-SMV | 11/3/13 | 40/27/1 | 0.056 |  |  | |  | |
| C-D >/= IIIa, yes/no (yes %) | 5/22 (18.5 %) | 13/55 (19.1 %) | 0.946 |  |  | |  | |
| Pancreatic fistula (Grade B or C), yes/no (yes %) | 1/26 (3.7 %) | 0/68 (0.0 %) | 0.284 |  |  | |  | |
| pPV positive, yes/no (yes%) | 1/26 (3.7 %) | 9/59 (13.2 %) | 0.160 |  |  | |  | |
| R0 resection, yes/no (yes %) | 23/4 (85.2 %) | 65/3 (95.6 %) | 0.098 |  |  | |  | |
| Postoperative hospital stays, days | **26 (15-69)** | **33 (14-95)** | **0.030** |  |  | |  | |

SVP: splenic vein preservation, SVR:splenic vein resection, SAL: splenic artery ligation, BMI: body mass index, UICC, R: resectable, BR: borderline resectable, UR: unresectable, NAC: neoadjuvant chemotherapy, NCRT: neoadjuvant chemoradiotherapy, PD: pancreaticoduodenectomy, SSPPD: subtotal stomach preserving PD, LGV: left gastric vein, IMV: inferior mesenteric vein, SV: splenic vein, C-D: Clavien-Dindo, pPV: pathological portal vein, R0 resection: curative resection
